# Supplementary material for: Cypsela and Pappus Morphology and Their Significance for the Taxonomic Delimitation of the Genus Saussurea DC. s.str. and Its Allied Genera (Asteraceae)
Source: Plants (Basel). 2024 Nov 29;13(23):3367. doi: 10.3390/plants13233367 (PMC11644217; doi:10.3390/plants13233367)
Supplement: Supplementary file 1 [file plants-13-03367-s001.zip › plants-3146421-supplementary.pdf]

Supplementary Table S1: Data matrix of the genus *Saussurea s.l.* for characters presented in Table 4 (From 1 to 88).

S.No.	Name of Taxa	1	2	3	4	5	6	7	8	9	10	11	12	13	14	15	16	17	18	19	20	21	22	23	24	25	26	27	28
1	*Saussurea obvallata*	3.5	1	1	0	0	0	0	0	0	0	0	0	0	0	0	0	0	0	0	0	0	0	0	0	0	1	0	1
2	*S. candolleana*	2.8	1	0	0	0	0	0	0	0	0	1	0	0	0	0	0	0	0	0	0	0	0	0	0	0	1	0	0
3	*S. falconeri*	3.9	1.2	1	0	0	0	0	0	0	0	0	0	0	0	0	0	0	0	0	0	0	0	0	0	1	0	0	0
4	*S. taraxacifolia*	4	1	0	0	0	0	0	0	0	0	0	0	1	0	0	0	0	0	0	0	0	0	0	0	0	1	0	1
5	*S. devendrae*	1.5	0.5	1	0	0	0	0	0	0	0	0	0	0	0	0	0	0	0	0	0	0	0	0	0	0	0	0	0
6	*S. andryaloides*	4	1.2	0	0	1	0	0	0	0	0	0	0	0	0	0	0	0	0	0	0	0	0	0	0	0	0	0	0
7	*S. atkinsonii*	3.9	2	0	0	0	0	0	0	0	0	0	0	0	1	0	0	0	0	0	0	0	0	0	0	0	0	0	0
8	*S. chondrilloides*	3.3	0.7	0	0	0	0	0	0	0	0	0	0	0	0	1	1	0	0	0	0	0	0	0	0	1	1	0	0
9	*S. leptophylla*	4.8	0.8	1	0	0	0	0	0	0	0	0	0	0	0	0	0	0	0	0	0	0	0	0	0	0	0	0	0
10	*S. simpsoniana*	2.5	0.8	1	0	0	0	0	0	0	0	0	0	0	0	0	0	0	0	0	0	0	0	0	0	0	0	0	0
11	*S. glacialis*	2.3	0.5	1	0	0	0	0	0	0	0	0	0	0	0	0	0	0	0	0	0	0	0	0	0	0	0	0	0
12	*S. gnaphalodes*	4	0.5	0	1	0	0	0	0	0	0	0	0	0	0	0	0	0	0	0	0	0	0	0	0	0	0	0	1
13	*S. bracteata*	1.4	0.8	0	0	0	0	0	0	0	0	1	0	0	0	0	0	0	0	0	0	0	0	0	0	0	0	0	0
14	*S. schultzii*	0.9	0.3	1	0	0	0	0	0	0	0	0	0	0	0	0	0	0	0	0	0	0	0	0	0	1	1	0	0
15	*S. elliptica*	3.8	1	1	0	0	1	0	0	0	0	0	0	0	0	0	0	0	0	0	0	0	0	0	0	0	0	0	1
16	*S. roylei*	5	1.3	1	0	0	0	0	0	0	0	0	0	0	0	0	0	0	0	0	0	0	0	0	0	0	1	0	1
17	*S. schlagintweitii*	4.5	1.2	0	0	1	0	0	0	0	0	0	0	0	0	0	0	0	0	0	0	0	0	0	0	0	0	0	1
18	*Lipschitziella. ceratocarpa var. ceratocarpa*	5.5	1.5	0	0	0	0	0	0	0	0	0	0	0	0	0	0	1	1	1	0	0	0	0	0	0	0	0	0
19	*L. ceratocarpa var. astorii*	7.8	1.8	0	0	0	0	0	0	0	0	0	0	0	0	0	0	1	1	1	0	0	0	0	0	0	0	0	1
20	*L. congesta* var. *pinnatisecta*	3.7	0.8	1	0	0	0	0	0	0	0	0	0	0	0	0	0	1	1	0	0	0	0	0	0	0	0	0	1
21	*L. congesta* var. *congesta*	3.6	0.7	1	0	0	0	0	0	0	0	0	0	0	0	0	0	1	1	1	0	0	0	0	0	0	0	0	0
22	*Dolomiaea megacephala*	4	2.8	0	0	0	0	0	0	0	1	0	0	0	0	0	0	0	0	0	0	0	0	0	0	1	0	0	0
23	*D. macrocephala*	6	1.5	0	0	0	0	0	0	0	0	0	1	0	0	0	0	0	0	0	0	0	0	0	0	0	1	0	0
24	*Shangwua jacea*	4.4	1	0	0	1	0	0	0	0	0	0	0	0	0	0	0	0	0	0	0	0	0	0	0	0	1	0	0
25	*Frolovia gilesii*	3.8	1.1	1	0	0	0	0	0	0	0	1	0	0	0	0	0	0	0	0	0	0	0	0	0	0	1	0	0
26	*Aucklandia costus*	9	1.4	0	0	0	0	1	0	0	0	0	0	0	0	0	0	0	0	0	0	0	0	0	0	0	1	1	0
27	*Himalaiella heteromalla*	3.8	1.3	1	0	0	0	0	0	0	0	0	0	0	0	0	0	0	0	0	1	0	0	0	0	0	0	0	0
28	*H. chitralica*	3	0.5	0	0	0	0	0	0	1	0	0	0	0	0	0	0	0	0	0	0	1	0	0	0	0	1	0	0
29	*H. afghana*	6	1.5	0	0	0	0	0	1	0	0	0	0	0	0	0	0	1	0	0	0	0	1	0	0	0	0	0	0
30	*H. albescens*	3.5	0.5	0	0	0	0	0	1	0	0	0	0	0	0	0	0	1	0	0	0	0	0	1	0	0	1	0	0
31	*H. chenopodifolia*	6	0.8	0	1	0	0	0	0	0	0	0	0	0	0	0	0	0	0	0	0	1	0	0	0	0	1	0	0
32	*H. diffusa*	3	1	0	0	0	0	0	1	0	0	0	0	0	0	0	0	0	0	0	0	0	0	0	1	0	1	0	0

S.No.	Name of Taxa	29	30	31	32	33	34	35	36	37	38	39	40	41	42	43	44	45	46	47	48	49	50	51	52	53	54
1	*Saussurea obvallata*	0	0	0	0	0	0	0	0	0	0	0	0	0	0	0	0	0	0	0	0	1	0	0	0	0	0
2	*S. candolleana*	0	0	1	0	0	0	0	0	0	0	0	0	0	0	0	0	0	0	0	0	0	1	1	0	0	0
3	*S. falconeri*	1	0	0	0	0	0	0	0	0	0	0	0	0	0	0	0	0	0	0	0	0	0	0	1	0	0
4	*S. taraxacifolia*	0	0	0	0	0	0	1	0	0	0	0	0	0	0	0	0	0	0	0	0	1	0	0	0	0	0
5	*S. devendrae*	0	0	0	0	0	0	0	0	0	0	1	0	0	0	0	0	0	1	0	0	0	0	0	0	1	0
6	*S. andryaloides*	0	0	0	0	0	0	0	0	0	0	0	0	0	0	0	0	0	0	0	1	0	0	0	0	0	1
7	*S. atkinsonii*	0	0	0	0	0	0	0	0	0	0	1	0	0	0	0	0	0	0	0	0	0	0	0	0	0	0
8	*S. chondrilloides*	1	0	0	0	1	0	0	0	0	0	0	0	0	0	0	0	0	0	0	0	0	0	0	0	0	0
9	*S. leptophylla*	0	1	0	1	0	0	0	0	0	0	0	0	0	0	0	0	0	0	0	0	0	0	0	0	0	0
10	*S. simpsoniana*	0	0	0	0	0	1	0	0	0	0	1	0	0	0	0	0	0	0	0	0	0	0	1	0	0	0
11	*S. glacialis*	0	0	0	0	0	0	0	0	1	0	0	0	0	0	1	0	0	0	0	0	0	0	0	0	0	0
12	*S. gnaphalodes*	0	0	0	0	0	0	1	0	0	0	0	0	0	0	0	0	0	0	0	0	0	0	0	0	0	0
13	*S. bracteata*	0	0	0	0	0	0	0	0	0	0	0	0	0	0	1	0	0	0	0	0	0	0	0	0	0	0
14	*S. schultzii*	0	0	0	0	0	0	0	0	0	0	1	0	0	0	0	0	0	0	0	0	0	0	1	0	0	0
15	*S. elliptica*	0	0	0	0	0	0	1	0	0	0	0	0	0	0	0	0	0	0	0	0	0	0	0	0	1	0
16	*S. roylei*	0	0	1	0	0	0	0	0	0	0	0	0	0	0	0	0	0	0	0	0	0	0	0	0	0	0
17	*S. schlagintweitii*	0	0	0	0	0	0	1	0	0	0	0	0	0	0	0	0	0	0	0	0	0	0	0	0	0	0
18	*Lipschitziella ceratocarpa var. ceratocarpa*	0	0	0	0	1	0	0	0	0	0	0	0	0	0	0	0	0	0	0	0	0	0	0	0	0	0
19	*L. ceratocarpa var. astorii*	0	0	0	0	0	0	0	0	0	0	0	0	0	0	0	0	0	0	0	0	0	0	0	0	0	0
20	*L. congesta* var. *pinnatisecta*	0	0	0	0	0	0	0	0	0	0	0	0	0	0	0	0	0	0	0	0	0	0	0	0	0	0
21	*L. congesta* var. *congesta*	0	0	0	0	0	0	0	0	0	1	0	0	0	0	0	0	0	0	0	0	0	0	0	0	0	0
22	*Dolomiaea megacephala*	0	0	0	0	0	0	0	0	0	0	0	0	0	0	0	0	1	0	1	0	0	0	0	0	0	0
23	*D. macrocephala*	0	0	0	0	0	0	0	0	0	0	0	0	0	0	0	0	0	0	0	0	0	0	0	0	0	0
24	*Shangwua jacea*	0	0	0	1	0	0	0	0	0	0	0	0	0	0	1	0	0	0	0	0	0	1	0	0	0	0
25	*Frolovia gilesii*	0	0	0	0	0	0	0	0	0	0	0	0	0	0	0	1	0	1	0	0	0	0	0	0	0	0
26	*Aucklandia costus*	0	0	1	0	0	0	0	1	0	0	0	0	0	0	0	0	0	0	0	0	0	0	0	0	0	0
27	*Himalaiella heteromalla*	0	0	0	0	0	0	0	0	0	0	1	1	0	0	0	0	0	0	0	0	0	0	0	0	0	0
28	*H. chitralica*	0	0	1	0	0	0	0	1	0	0	0	0	0	0	0	0	0	0	0	0	0	0	0	0	0	0
29	*H. afghana*	0	0	0	1	0	0	0	0	0	0	0	1	0	0	1	0	0	0	0	0	0	0	0	0	0	0
30	*H. albescens*	0	0	0	0	0	0	0	0	0	0	0	0	1	0	0	0	0	0	0	0	0	0	0	0	0	0
31	*H. chenopodifolia*	0	0	0	0	0	0	0	0	0	0	0	0	0	1	0	0	0	0	0	0	1	0	0	0	0	0
32	*H. diffusa*	0	0	0	0	1	0	0	0	0	0	0	0	0	0	0	0	0	0	0	0	0	0	0	0	0	0

S.No.	Name of Taxa	55	56	57	58	59	60	61	62	63	64	65	66	67	68	69	70	71	72	73	74	75	76	77	78
1	*Saussurea obvallata*	0	0	0	0	0	0	0	0	1	0	9	0	0	1	0	0	0	0	0	0	0	0	0	1
2	*S. candolleana*	0	0	0	0	0	0	0	0	1	0	9	0	0	0	1	0	0	0	0	0	0	0	0	1
3	*S. falconeri*	0	0	0	0	0	0	0	0	1	0	14.5	1	0	0	0	0	0	0	0	0	0	0	0	1
4	*S. taraxacifolia*	0	0	0	0	0	0	0	0	1	0	17.5	0	0	0	0	1	0	0	0	0	0	0	0	1
5	*S. devendrae*	0	0	0	0	0	0	0	0	1	0	7	0	0	0	1	0	0	0	0	0	0	0	0	1
6	*S. andryaloides*	0	0	0	0	0	0	0	0	1	0	9	0	0	0	0	1	0	0	0	0	0	0	1	0
7	*S. atkinsonii*	1	0	0	0	0	0	0	0	1	0	9.5	0	0	0	0	1	0	0	0	0	0	0	0	0
8	*S. chondrilloides*	1	0	0	0	0	0	0	0	1	0	7	0	0	0	0	1	0	0	0	0	0	0	1	0
9	*S. leptophylla*	0	1	0	0	0	0	0	0	1	0	7.5	0	0	0	0	0	1	0	0	0	0	0	0	1
10	*S. simpsoniana*	0	0	0	0	0	0	0	0	1	0	13	0	0	0	0	1	0	0	0	0	0	0	0	1
11	*S. glacialis*	1	0	0	0	0	0	0	0	1	0	9	0	0	0	1	0	0	0	0	0	0	0	0	1
12	*S. gnaphalodes*	1	0	0	0	0	0	0	0	1	0	9.5	0	1	0	0	0	0	0	0	0	0	0	0	1
13	*S. bracteata*	1	0	0	0	0	0	0	0	1	0	8.5	0	0	0	0	0	0	1	0	0	0	0	0	1
14	*S. schultzii*	0	0	0	0	0	0	0	0	1	0	4	0	0	0	0	0	0	1	0	0	0	0	0	1
15	*S. elliptica*	0	0	0	0	0	0	0	0	1	0	12.5	0	0	0	0	1	0	0	0	0	0	0	0	1
16	*S. roylei*	1	0	0	0	0	0	0	0	1	0	11.5	0	1	0	0	0	0	0	0	0	0	0	0	1
17	*S. schlagintweitii*	0	1	0	0	0	0	0	0	1	0	13	0	1	0	0	0	0	0	0	0	0	0	0	1
18	*Lipschitziella ceratocarpa var. ceratocarpa*	0	0	0	0	0	1	0	1	0	0	10	0	0	0	0	0	0	1	0	0	0	0	1	0
19	*L. ceratocarpa var. astorii*	0	0	0	0	0	1	0	1	0	0	11	0	0	0	0	0	0	1	0	0	0	0	0	1
20	*L. congesta* var. *pinnatisecta*	0	0	0	0	0	1	0	1	0	0	11.5	0	0	0	0	0	0	1	0	0	0	0	0	0
21	*L. congesta* var. *congesta*	0	0	0	0	0	1	0	1	0	0	11.5	0	0	0	0	0	0	1	0	0	0	0	0	1
22	*Dolomiaea megacephala*	0	0	0	0	1	0	0	0	0	1	29	0	0	0	0	0	0	0	0	1	0	0	0	1
23	*D. macrocephala*	0	0	0	0	0	0	0	0	0	1	16	0	0	0	0	0	0	0	1	0	0	0	0	0
24	*Shangwua jacea*	0	0	0	0	0	0	0	1	0	0	1.5	0	0	0	0	0	0	0	0	0	1	0	0	1
25	*Frolovia gilesii*	0	0	0	0	0	0	1	1	0	0	14.5	0	0	0	0	0	0	0	0	0	0	1	0	1
26	*Aucklandia costus*	0	0	1	0	0	0	0	1	0	0	1.4	0	0	0	0	0	0	0	0	0	0	0	0	1
27	*Himalaiella heteromalla*	0	0	0	1	0	0	0	1	0	0	13	0	0	0	0	0	1	0	0	0	0	0	0	1
28	*H. chitralica*	1	0	0	0	0	0	0	1	0	0	12	0	0	0	0	0	1	0	0	0	0	0	0	0
29	*H. afghana*	1	0	0	0	0	0	0	1	0	0	13	0	0	0	0	0	1	0	0	0	0	0	1	0
30	*H. albescens*	1	0	0	0	0	0	0	1	0	0	10	0	0	0	0	0	0	0	1	0	0	0	0	0
31	*H. chenopodifolia*	0	0	0	0	0	0	0	1	0	0	11	0	0	0	0	0	1	0	0	0	0	0	0	0
32	*H. diffusa*	1	0	0	0	0	0	0	1	0	0	11	0	0	0	0	0	1	0	0	0	0	0	1	0

S.No.	Name of Taxa	79	80	81	82	83	84	85	86	87	88
1	*Saussurea obvallata*	0	1	1	0	0	0	0	0	0	0
2	*S. candolleana*	0	1	0	1	0	0	0	0	0	0
3	*S. falconeri*	0	1	0	0	1	0	0	0	0	0
4	*S. taraxacifolia*	0	1	0	0	0	1	0	0	0	0
5	*S. devendrae*	1	0	1	0	0	0	0	0	0	0
6	*S. andryaloides*	0	0	0	0	0	0	0	0	0	0
7	*S. atkinsonii*	0	0	0	0	0	0	0	0	0	0
8	*S. chondrilloides*	0	0	0	0	0	0	0	0	0	0
9	*S. leptophylla*	1	0	0	0	0	0	1	0	0	0
10	*S. simpsoniana*	1	0	0	0	0	0	1	0	0	0
11	*S. glacialis*	1	0	0	0	0	0	0	0	0	0
12	*S. gnaphalodes*	1	0	0	0	0	0	1	1	0	0
13	*S. bracteata*	1	0	0	0	0	0	0	0	1	0
14	*S. schultzii*	0	1	0	0	0	1	0	0	0	0
15	*S. elliptica*	1	0	0	0	0	0	0	0	1	0
16	*S. roylei*	1	0	0	0	0	0	0	0	0	1
17	*S. schlagintweitii*	1	0	0	0	0	0	1	0	0	0
18	*Lipschitziella ceratocarpa var. ceratocarpa*	0	0	0	0	0	0	0	0	0	0
19	*L. ceratocarpa var. astorii*	0	1	0	0	0	0	0	0	0	1
20	*L. congesta* var. *pinnatisecta*	0	0	0	0	0	0	0	0	0	0
21	*L. congesta* var. *congesta*	0	1	0	0	0	0	0	0	0	1
22	*Dolomiaea megacephala*	1	0	0	0	0	1	0	0	0	0
23	*D. macrocephala*	0	0	0	0	0	0	0	0	0	0
24	*Shangwua jacea*	1	0	0	0	0	0	0	0	0	1
25	*Frolovia gilesii*	1	0	1	0	0	0	0	0	0	0
26	*Aucklandia costus*	1	0	0	0	0	0	0	0	0	1
27	*Himalaiella heteromalla*	0	1	0	0	0	0	0	0	0	1
28	*H. chitralica*	0	0	0	0	0	0	0	0	0	0
29	*H. afghana*	0	1	0	0	0	0	0	0	0	0
30	*H. albescens*	0	0	0	0	0	0	0	0	0	0
31	*H. chenopodifolia*	0	0	0	0	0	0	0	0	0	0
32	*H. diffusa*	0	0	0	0	0	0	0	0	0	0
